# Five Withanolides from the Leaves of *Datura metel* L. and Their Inhibitory Effects on Nitric Oxide Production

**DOI:** 10.3390/molecules19044548

**Published:** 2014-04-11

**Authors:** Bing-You Yang, Rui Guo, Ting Li, Yan Liu, Chang-Fu Wang, Zun-Peng Shu, Zhi-Bin Wang, Jing Zhang, Yong-Gang Xia, Hai Jiang, Qiu-Hong Wang, Hai-Xue Kuang

**Affiliations:** Key Laboratory of Chinese Materia Medica (Ministry of Education), Heilongjiang University of Chinese Medicine, Ministry of Education, Harbin 150040, China

**Keywords:** *Datura metel* L., withanolides, dmetelins, NO inhibition

## Abstract

Four new withanolides named dmetelins A–D (compounds **1**–**4**), along with the known compound 7*α*,27-dihydroxy-1-oxo-witha-2,5,24-trienolide (**5**) were isolated from the leaves of *Datura metel* L. (Solanaceae). Their structures were elucidated on the basis of detailed analysis of 1D and 2D NMR and mass spectrometry data. All the compounds were evaluated for their inhibitory effects on lipopolysaccharide (LPS)-induced nitric oxide (NO) production in RAW 264.7 cells. Compounds **1**, **4** and **5** showed significant inhibitory activities, and compounds **2** and **3** showed moderate inhibitory activities with IC_50_ values of 17.8, 11.6, 14.9, 33.3 and 28.6 μM, respectively.

## 1. Introduction

Withasteroids are a group of structurally diverse steroidal compounds with a C_28_ steroidal lactone skeleton, in which a characteristic feature is the presence of an *α*,*β*-unsaturated *δ* lactone ring in the side chain. They are presented primarily in the Solanaceae family, which includes *Datura*, *Acnistus*, *Dunalia*, *Jaborosa*, *Physalis* and *Withania* [1]. The isolation and synthesis of withanolides have received considerable attention due to their significant biological activities, which include antitumor [2], cytotoxic [3,4], immunosuppressive [4], anti-inflammatory [5,6], and chemoprevention properties [7].

**Figure 1:**
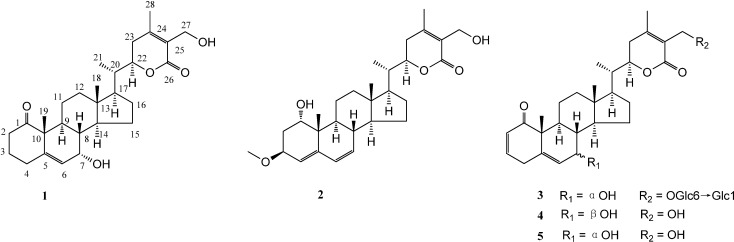
Structures of compounds **1**–**5**.

Flos daturae (baimantuoluo in Chinese), the dry flowers of *Datura metel* L. (Solanaceae), known as “Yangjinhua”, have been widely used in Traditional Chinese Medicine for the treatment of coughs, asthma, rheumatism, pain, and convulsions for centuries [8]. It has also been reported that it displayed the most promising effects in treatment of psoriasis and were used in a clinical application at the First Affiliated Hospital of Heilongjiang University of Chinese Medicine (Heilongjiang, China) [9,10]. Withanolides have been studied for treating psoriasis as the main constituents of the effective part of flowers of *D. metel* [11,12]. Baimantuoluolines A–J, and baimantuoluosides A–H were also isolated and reported [8,13,14,15,16,17,18]. However, the dry flowers of *D. metel* have the disadvantages of long florescence and low yield in comparison to its leaves. At the same time, the leaves of this herb, which can be regenerated every year, were typically discarded. In order to expand the available resources, our group found that the leaves of *D. metel* have some similarities in chemical constituents with its flowers and significant advantages in terms of high and stable yield. As a result, four new withanolides were isolated and named dmetelins A–D (compounds **1**–**4**), together with one known withanolide, 7*α*,27-dihydroxy-1-oxowitha-2,5,24-trienolide (**5**) [19] (Figure 1). The structures of compounds **1**–**5** were determined by the interpretation of spectroscopic analysis, including 1D and 2D NMR spectroscopy. All isolates were identified as major active constituents having inhibitory effects of NO production in LPS-activated macrophage cell line, RAW 264.7 murine macrophages. Herein, we report the isolation, structural elucidation, and NO inhibitory effects of these isolates.

## 2. Results and Discussion

**Figure 2:**
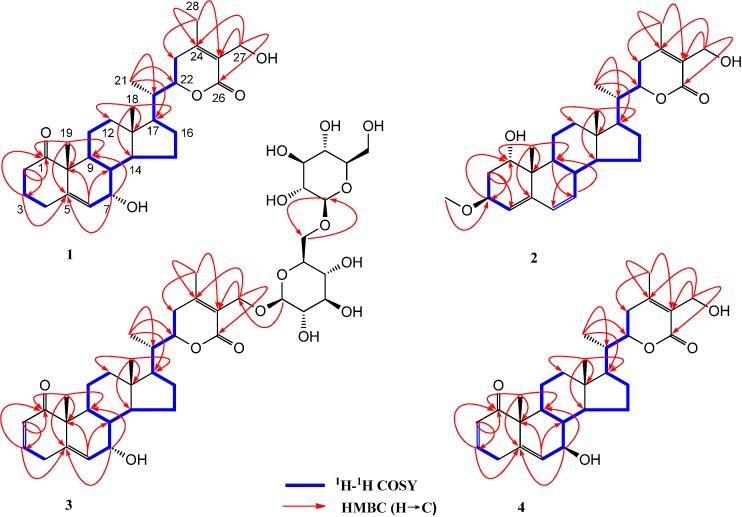
^1^H, ^1^H-COSY and Selected HMBC correlations of compounds **1**–**4**.

Compound **1** was isolated as a white amorphous powder, and its molecular formula was determined to be C_28_H_40_O_5_ by HRESIMS (*m/z* 479.2779 [M+Na]^+^). The UV spectrum showed an absorption maximum at 224 nm, suggesting the presence of an *α*,*β*-unsaturated *δ* lactone, and carbonyl groups, respectively [20]. The ^1^H-NMR spectrum of **1** (Table 1) displayed the characteristic signals of the common withanolide steroid. Three tertiary methyl groups at *δ* 0.78 (3H, *s*), 1.28 (3H, *s*), 2.10 (3H, *s*), and a secondary methyl group at 1.04 (3H, *d*, *J* = 6.6 Hz) were attributed to Me-18, Me-19, Me-28, and Me-21, respectively. The Me-27 signal was replaced by one set of oxygen-bearing methylene protons *δ* 4.37 (1H, *d*, *J* = 11.7 Hz) and 4.29 (1H, *d*, *J* = 11.7 Hz). An olefinic proton at *δ* 5.70 (1H, *dd*, *J* = 5.3, 1.2 Hz) showed ^1^H-^1^H COSY correlation peaks with H-7 *δ* 3.77 (1H, *t*, *J* = 3.8 Hz) (Figure 2), which was assigned to the vinylic protons H-6. The ^13^C-NMR spectrum of **1** (Table 2) revealed 28 carbons. It was composed of four methyl groups at *δ* 12.1 (C-18), 18.7 (C-19), 13.8 (C-21), and 20.2 (C-28). The presence of four olefin carbons at *δ* 146.6, 125.9, 157.9, and 126.4 were attributed to C-5, C-6, C-24, and C-25, respectively. The characteristic downfield at *δ* 215.9 and 168.6 were due to a carbonyl group C-1 and a lactone carbonyl group C-26. The signals at *δ* 65.1, 80.2, and 56.4 were assigned to the three oxygen-bearing carbons at C-7, C-22, and C-27. Assignments of all function groups of **1** were achieved by ^1^H-^1^HCOSY, HMBC and HSQC (Figure 2). Thus, the planar structure of **1** was determined as shown.

**Table 1:** ^1^H-NMR data (400 MHz) of the aglycones of **1**–**4** (in CD_3_OD, *δ* in ppm).

No.	1	2	3	4
1		3.88 br s		
2	2.32 m, 2.67 m	1.85 m, 2.24 m	5.86 dd (10.0, 2.4)	5.84 dd (10.0, 2.5)
3	2.00 m, 1.62 m	4.11 t (7.4)	6.93 ddd (10.0, 4.8, 2.4)	6.91 ddd (10.0, 4.8, 2.5)
4	2.22 m, 2.63 m	5.48 br s	3.44 m	3.40 m
			2.96 dd (21.5, 4.8)	2.93 dd (21.4, 4.8)
6	5.70 dd (5.3, 1.2)	5.98 dd (10.0, 2.3)	5.80 dd (5.8, 1.5)	5.48 br s
7	3.77 t (3.8)	5.63 br d (10.0)	3.80 t (5.5)	3.71 d (8.4)
8	1.43 m	2.07 m	1.46 m	1.42 m
9	1.99 m	1.54 m	2.00 m	1.68 m
11	1.50 m, 1.73 m	1.44 m, 1.66 m	1.59 m, 2.23 m	1.53 m, 2.24 m
12	1.29 m, 1.97 m	1.32 m, 2.03 m	1.34 m, 2.03 m	1.32 m, 2.04 m
14	1.54 m	1.24 m	1.32 m	1.24 m
15	1.78 m, 1.19 m	1.85 m, 1.37 m	1.84 m, 1.22 m	1.92 m, 1.53 m
16	1.79 m, 1.41 m	1.83 m, 1.43 m	1.83 m, 1.41 m	1.78 m, 1.37 m
17	1.24 m	1.30 m	1.28 m	1.22 m
18	0.78 s	0.82 s	0.79 s	0.80 s
19	1.28 s	1.00 s	1.24 s	1.28 s
20	1.95 m	1.98 m	1.98 m	1.94 m
21	1.04 d (6.6)	1.03 d (6.7)	1.05 d (6.6)	1.04 d (6.6)
22	4.47 dt (13.2, 3.4)	4.47 dt (13.3, 3.4)	4.50 dt (13.5, 3.6)	4.48 dt (13.2, 3.4)
23	2.56 dd (17.8, 13.7)	2.54 dd (18.0, 13.4)	2.58 dd (17.9, 13.6)	2.54 dd (18.0, 13.5)
	2.18 dd (17.8, 3.4)	2.21 dd (18.0, 3.3)	2.22 dd (17.9, 3.0)	2.20 dd (18.0, 3.1)
27	4.29 d (11.7)	4.36 d (11.7)	4.47 d (11.2)	4.37 d (11.7)
	4.37 d (11.7)	4.29 d (11.7)	4.62 d (11.2)	4.29 d (11.7)
28	2.10 s	2.10 s	2.14 s	2.10 s
**OCH_3_**		3.38 s		

**Table 2:** ^13^C-NMR (100 MHz) data of the aglycones of **1**–**4** (in CD_3_OD, *δ* in ppm).

No.	1	2	3	4
1	215.9	72.5	205.7	205.9
2	39.0	33.0	128.4	128.4
3	26.2	75.4	147.7	147.6
4	31.9	123.4	34.4	33.8
5	146.6	143.3	141.7	138.2
6	125.9	130.1	127.5	130.8
7	65.1	132.1	64.8	72.1
8	38.6	38.5	39.5	42.2
9	36.2	45.3	36.4	42.6
10	55.5	40.4	52.3	51.4
11	23.3	21.4	24.6	24.9
12	40.5	40.9	40.8	41.0
13	43.7	45.1	43.6	44.4
14	50.7	55.2	51.0	57.4
15	25.1	25.0	25.0	27.7
16	28.3	28.3	28.2	28.5
17	53.2	53.2	53.2	52.7
18	12.1	12.2	12.2	12.4
19	18.7	19.9	18.8	19.2
20	40.5	40.4	40.5	40.4
21	13.8	13.7	13.8	13.8
22	80.2	80.1	80.2	80.2
23	30.7	30.7	30.8	30.7
24	157.9	157.9	160.4	157.9
25	126.4	126.4	123.6	126.4
26	168.6	168.5	168.6	168.6
27	56.4	56.4	63.6	56.4
28	20.2	20.2	20.9	20.2
OCH_3_		55.8		

**Figure 3:**
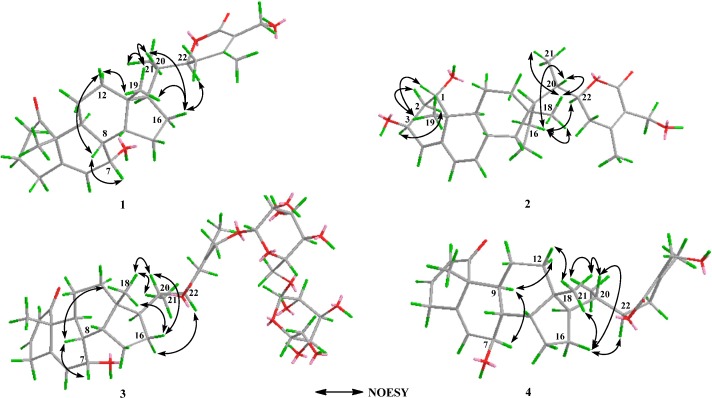
Key NOESY contacts of compounds **1**–**4**.

The configuration of compound **1** (Figure 3) was established by NOE correlations. Me-18/Me-21, H-20, H-16*β*, H-20/H-16*β*, and H-16/H-22 determined that C-20 of **1** had an *S* configuration. According to the literature [3,18], an *α*-oriented H-22 atom gives rise to H-22/H-23 values of 0.5–4.0 and 9.0–13.8 Hz, whereas the *β*-oriented form shows values of 2.5-7.0 and 2.0-5.0 Hz. Thus, the configuration of **1** at C-22 was determined as *R* due to the H-22 coupling constants (*J* = 3.4, 13.2 Hz). The orientation of hydroxyl group at C-7 was also deduced from the NOESY spectrum (Figure 3). The NOE correlations of Me-18/H-8*β* and H-8*β*/H-7*β* suggested the *β*-orientation of H-7 and *α* orientation of the 7-OH group. On the basis of the spectroscopic studies, the structure of **1** was fully established, and determined to be 7*α*,27-dihydroxy-(20*S*,22*R*)-1-oxo-witha-5,24-dienolide and given the trivial name dmetelin A.

Compound **2** was obtained as a white amorphous powder with the molecular formula C_29_H_42_O_5_, established by positive HRESIMS from the [M+Na]^+^ signal at *m/z* 493.2921. The NMR data of **2** exhibited a close resemblance to those of **1**, and differences between them were observed only in their A/B rings. The NMR data of **1** showed a conjugated diene system composed of a disubstituted double bond (*δ* 5.98 and 130.1, *δ* 5.63 and 132.1), together with one trisubstituted double bond (*δ* 5.48, 143.3, and 123.4). The presence of an oxygenated methine at *δ* 72.5 (C-1) instead of the keto carbon in **1** implied that a hydroxyl group was attached to C-1. The HMBC correlations of *δ* 2.24 (H-2)/123.4, 2.07 (H-8)/130.1, and 1.54 (H-9)/132.1 suggested that the conjugated diene system was located at C-4, C-5, C-6, and C-7 (Figure 2). A three proton singlet at *δ* 3.38 (3H, *s*) was due to -OCH_3_ group, which was located at C-3 on the basis of *δ* 3.38/75.4 (C-3) observed in its HMBC spectrum (Figure 2). The orientation of the hydroxyl group at C-1 was assigned as *α* on the basis of the small coupling constants of H-1 *δ* 3.88 (1H, *br s*)/H-2. Furthermore, the NOESY correlation of Me-19/H-1*β* also supported the *α* orientation of 1-OH. In addition, NOEs between Me-19/H-2*β* and H-2*α*/H-3*α* suggested a *β* orientation of 3-OCH_3_. The configuration of C-20 and C-22 were in good agreement with that of **1**. Consequently, compound **2** was identified as 1*α*,3*β*,27-trihydroxy-(20*S*,22*R*)-1-oxo-witha-4,6,24-trienolide and given the common name dmetelin B.

Compound **3** was isolated as a white amorphous powder. The HRESIMS spectrum showed peaks at 801.3694 [M+Na]^+^, corresponding to the molecular formula C_40_H_58_O_15_. The NMR spectra (Table 1 and Table 2) of **3** showed four methyl groups at *δ* 0.79 (3H, *s*, Me-18), 1.24 (3H, *s*, Me-19), 1.05 (3H, *d*, *J* = 6.6 Hz, Me-21), and 2.14 (3H, *s*, Me-28), two *α*, *β*-unsaturated olefinic protons at *δ* 5.86 (1H, *dd*, *J* = 10.0, 2.4 Hz, H-2) and 6.93 (1H, *ddd*, *J* = 10.0, 4.8, 2.4 Hz, H-3), along with one olefinic proton at *δ* 5.80 (1H, *dd*, *J* = 5.8, 1.5 Hz, H-6), which supported a withanolide possessing a 2, 5-dien-1-one system in rings A/B [21]. Apart from the glycosidic signals, compound **3** displayed 28 carbons. The signals at *δ* 160.4, 123.6 and 168.6 were assigned to C-24, C-25 and C-26 of the *α*, *β*-unsaturation *δ* lactone ring respectively, in the six-member ring E. Two anomeric protons doublets at *δ* 4.34 (1H, *d*, *J* = 7.8 Hz, H-1′) and 4.41 (1H, *d*, *J* = 7.8 Hz, H-1″) were correlated to two anomeric carbons at *δ* 103.9 (C-1′) and 104.9 (C-1″), in its HSQC spectrum (Figure 2), which suggested that **3** contained a diglycosidic moiety. The attachment of one glucose unit to C-27 of the aglycone was confirmed by the HMBC correlations (Figure 2) observed from H-1′ to C-27. Other HMBC correlations to glucose linkages (Figure 2) were H-1″/C-6′ and H-6′/C-1″. The orientation of 7-OH could be deduced by Me-18/H-8*β* and H-8*β*/H-7*β* observed in the NOESY spectrum, which suggested that 7-OH has an *α* orientation. The configuration of C-20 and C-22 of **3** were established to be identical to those of **1** by their similar NOESY correlations, as depicted on the 3D structure (Figure 3). Hence, **3** was identified as 7*α*,27-dihydroxy-(20*S*,22*R*)-1-oxo-witha-2,5,24-trienolide-27-*O*-*β*-d-glucopyranosy-(6′→1″)-*β*-d-glucopyranoside and assigned the common name dmetelin C.

Compound **4** was obtained as a white amorphous powder. The molecular formula C_28_H_38_O_5_ was deduced from the HRESIMS with [M+H]^+^ at *m/z* 455.2715 (calcd. for C_28_H_3__9_O_5_, 455.2797). The NMR data of **4** (Table 1 and Table 2) were very similar to those of 7*α*,27-dihydroxy-1-oxowitha-2,5,24-trienolide [19], also isolated as compound **5** during this work. A comparison of the ^13^C-NMR data of these two compounds indicated that they have the same planar structure. The key differences were the signals for carbons around C-7, which suggested that these two compounds should have different orientations of the hydroxyl group at C-7. The orientation of 7-OH was deduced by the NOE observed for Me-18/H-12*β*, H-12*α*/H-9*α*, and H-9*α*/H-7*α* in the NOESY experiment (Figure 3), indicating the *α*-orientation of H-7 and *β*-orientation of the 7-OH group. The configuration of C-20 and C-22 were also akin to those of compound **1**. Therefore, the structure of **4** was identified as 7*β*,27-dihydroxy-(20*S*,22*R*)-1-oxo-witha-2,5,24-trienolide and given the common name dmetelin D.

**Figure 4:**
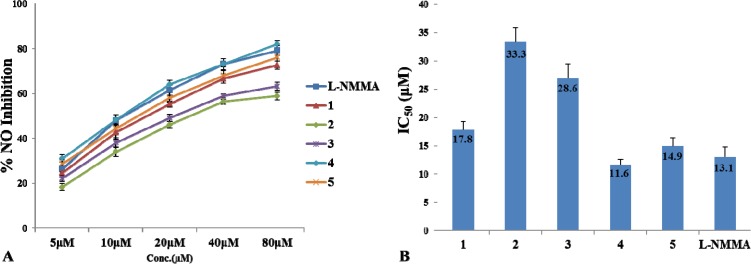
Inhibitory activities of compounds **1**–**5** on NO production in LPS-induced RAW 264.7 cells. (**A**) Inhibitory effects on NO production of compounds **1**–**5** and positive control (l-NMMA). All results are expressed as the mean ± S.D. of three independent experiments, each one performed in triplicate. (**B**) IC_50__s_ of compounds **1**–**5** and the positive control.

Since NO acts as an inflammatory mediator, inhibitors of NO production may exhibit therapeutic potential for the treatment of inflammation with overproduction of NO. Withanolides display great anti-inflammatory activity [22,23,24], so the compounds were evaluated for their effects on the inhibition of NO production in LPS-activated RAW264.7 cells [25]. In our experiment, the assayed compounds were first dissolved in DMSO, and the final concentration 0.2% (v/v) in cell culture supernatants did not show any effect on the assay systems. The effect of compounds **1**–**5** on cell viability was evaluated by the MTT assay to ascertain the absence of cytotoxicity (over 90% cell survival) to macrophage cells at the concentration of 100 μM. The inhibitory effects of withanolides (Figure 4A) were evaluated by LPS-induced NO generation in RAW 264.7 cells. Compounds **1**, **4** and **5** presented the significant anti-inflammatory activity, with IC_50_ values of 17.8, 11.6 and 14.9 μM, respectively. l-NMMA (IC_50_ = 13.1 μM) was used as positive control. Compounds **2** and **3** revealed moderate anti-inflammatory activity, with IC_50_ values of 33.3 and 28.6 μM (Figure 4B).

## 3. Experimental Section

### 3.1. General

Optical rotations were measured using a Perkin-Elmer 341 polarimeter. UV spectra were obtained on a Shimadzu UV-1601 instrument. IR spectra were recorded on a Shimadzu FTIR-8400S. NMR spectra were recorded in MeOH using TMS as an internal standard on Bruker DPX 400 instrument (400 MHz for ^1^H NMR and 100 MHz for ^13^C NMR). Chemical shifts (*δ*) were expressed in ppm with reference to the solvent signals. HRESIMS was recorded on IonSpec Ultima 7.0 FTICR; GC analysis was performed on Agilent 7890A-5975C gas chromatograph equipped with a DB-1701 column (60 m × 0.25 mm × 0.25 μm, film thickness); detection Triple-Axis Detector; Preparative HPLC (Waters, Delta 600-2487) was measured by Hypersil-ODS II (10 μm, 20 × 300 mm, Yilite, Da Lian, China); Column chromatography (CC) was carried out using silica gel of 200–300 mesh size (Qingdao Marine Chemical Ltd, Qingdao, China); All the solvents were of analytical grade and were purchased from TianJinFuYu Company Ltd. (Tianjin, China)

### 3.2. Plant Material

The leaves of *D. metel* were collected at Lingao County, Hainan Province, People’s Republic of China, in September 2012, and was identified by Prof. Zhenyue Wang (Department of Chinese Medicine Resources, Heilongjiang University of Chinese Medicine). A voucher specimen (2012184) has been deposited at the Herbarium of Heilongjiang University of Chinese Medicine, P. R. China.

### 3.3. Extraction and Isolation

The air-dried leaves of *D. metel* (16 kg) were extracted with 75% EtOH (100 L × 3.0 h × 3) at room temperature. The 75% EtOH extract was evaporated under vacuum (45 °C) to yield a residue (1.75 kg), which was suspended in water and then sequentially extracted with petroleum ether (PE) and EtOAc. The EtOAc portion of the extract (226 g) was chromatographed on silica gel with CH_2_Cl_2_/MeOH (30:1 → 0:1, v/v) to afford Fractions A–E. Fraction B (36.2 g) was rechromatographed on a silica gel column (CH_2_Cl_2_/MeOH 25:1 → 0:1, v/v) to give five subfractions (B1-B5). Compound **5** (22 mg) was obtained from fraction B2 after purification by CC using (CH_2_Cl_2_/MeOH 20:1 → 0:1, v/v), Subfraction B3 was further purified by ODS column, eluting with MeOH/H_2_O (1:9 → 1:0, v/v) to yield **4** (28 mg). Subfraction B4 was chromatographed over a ODS column (MeOH/H_2_O 2:8 → 1:0, v/v) and semi-preparative HPLC (66% MeOH/H_2_O, flow rate 3 mL/min) to give **1** (18 mg, *t*_R_ = 39.1 min), and **2** (15 mg, *t*_R_ = 45.3 min). Fraction E (11.6 g) was purified by CC and eluted with CH_2_Cl_2_/MeOH (10:1 → 0:1, v/v) to afford three subfractions (E1–E3), and Subfraction E3 was further purified by semi-preparative HPLC (40% MeOH/H_2_O, flow rate 3 mL/min) to yield **3** (15 mg, *t*_R_ = 25.6 min).

*Dmetelin A* (**1**). 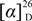 + 28.0 (*c* = 0.10, MeOH); UV (MeOH) λ_max_ (logε): 224 (4.37) nm; IR (KBr) *v*_max_: 3401, 3169, 2933, 2688, 1701, 1689, 1262, 1068, 1023, 821 cm^−1^; ^1^H and ^13^C-NMR data, see Table 1 and Table 2; HRESIMS *m/z*: 479.2779 [M+Na]^+^ (calcd. for C_28_H_40_O_5_Na, 479.2773).

*Dmetelin B* (**2**). 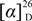 + 16.0 (*c* = 0.10, MeOH); UV (MeOH) λ_max_ (logε): 225 (5.60) nm; IR (KBr) *v*_max_: 3367, 2963, 2869, 1685, 1388, 1075 cm^−1^; ^1^H and ^13^C-NMR data, see Table 1 and Table 2; HRESIMS *m/z* 493.2921 [M+Na]^+^ (calcd. for C_29_H_42_O_5_Na, 493.2930).

*Dmetelin C* (**3**). 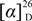 + 44.0 (*c* = 0.10, MeOH); UV (MeOH) λ_max_ (logε): 222 (5.28) nm; IR (KBr) *v*_max_: 3400, 2937, 2360, 2349, 1716, 1695, 1681, 1219, 1126, 1028 cm^−1^; ^1^H and ^13^C-NMR data, see Table 1, Table 2, and Table 3; HRESIMS *m/z* 801.3694 [M+Na]^+^ (calcd. for C_40_H_58_O_15_Na, 801.3673).

*Dmetelin D* (**4**). 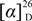 + 20.0 (*c* = 0.10, MeOH); UV (MeOH) λ_max_ (logε): 221 (6.52) nm; IR (KBr) *v*_max_: 3402, 3367, 3129, 2963, 2915, 2869, 1685, 1075, 799 cm^−1^; ^1^H and ^13^C-NMR data, see Table 1 and Table 2; HRESIMS *m/z* 455.2715 [M+H]^+^ (calcd. for C_28_H_3__9_O_5_, 455.2797).

**Table 3:** ^1^H- and ^13^C-NMR data (400/100 MHz) data of the glycosyl groups for **3** (in CD_3_OD, *δ* in ppm).

NO.	δ_H_	δ_C_		NO.	δ_H_	δ_C_
1′	4.34 d (7.8)	103.9		1″	4.41 d (7.8)	104.9
2′	3.18 t (8.9)	75.2		2″	3.36 t (9.2)	75.0
3′	3.41 m	77.9		3″	3.30 m	78.0
4′	3.28 m	71.7		4″	3.36 m	71.5
5′	3.43 m	77.2		5″	3.30 m	78.0
6′	3.78 dd (11.6, 5.8)	69.9		6″	3.68 dd (11.8, 5.0)	62.8
	4.16 dd (11.6, 1.8)				3.86 dd (11.8, 2.0)	

### 3.4. Acid Hydrolysis of Compound **3** and GC Analysis

Compound **3** (2.0 mg) was refluxed with H_2_O (2 mL) and 2 N aqueous HCl (1 mL) at 80 °C on a water bath for 3 h. After that time, the reaction mixture was extracted with ethyl acetate (3 × 5 mL). The aqueous layer was neutralized with 2 M NaHCO_3_ and then evaporated to dryness. The residue of sugar was dissolved in 1 mL anhydrous pyridine and treated with l-cysteine methyl ester hydrochloride (1.5 mg) stirred at 60 °C for 1 h. HMDS-TMCS (150 μL hexamethyldisilazane-trimethylchlorosilane, 3:1) was added and the mixture was kept at 60 °C for another 30 min. The precipitate was centrifuged off, and the supernatant was concentrated under a N_2_ stream. The mixture was partitioned between *n*-hexane and H_2_O (0.1 mL each), and the hexane extracted (1 μL) was analyzed on an Agilent 7890A-5975C gas chromatograph equipped with a DB-1701 column (60 m × 0.25 mm × 0.25 μm, film thickness) under the following conditions: firstly temperature was maintained at 50 °C, secondly raising to 190 °C at the rate of 40 °C/min, thirdly raising the temperature to 200 °C at the rate of 0.5 °C/min, fourthly raising the temperature to 210 °C at the rate of 1 °C/min, finally, raising the temperature to 280 °C at the rate of 20 °C/min. The carrier gas was He (1.0 mL/min), injector temperature: 250 °C; and split ratio: 1/20. By comparison of the retention times of authentic samples of d-glucose, the absolute configurations of the sugar residues were gave to be d-glucose (*t*_R_ = 17 min).

### 3.5. Cell Culture

Raw 264.7, a murine macrophage cell line was purchased from the Cell Bank of the Chinese Academic of Sciences, (Shanghai, China) and maintained in supplemented with 10% Fetal Bovine Serum (Hyclone, Logan City, UT, USA), penicillin (100 U/mL) and streptomycin (100 μg/mL) at 37 °C in a humidified incubator with 5% CO_2_.

### 3.6. Cell Viability Assay

Cell viability was assessed using the MTT assay as described previously [26,27]. In brief, RAW 264.7 cells were seeded into a 96-well plate at a density of 1.0 × 10^5^ cells per well and incubated at 37 °C for 24 h. The cells were then treated with various concentrations of the samples. The maximum concentration of vehicle (dimethylsulfoxide, DMSO) in the culture media was 0.2% (v/v). After an additional 24 h incubation at 37 °C, 100 μL of MTT (0.5 mg/mL in PBS) was added to the wells, and the incubation continued for another 2 h. The medium was then discarded and 150 μL DMSO was added. The resulting color was assayed at 540 nm using a microplate reader (Molecular Devices, San Francisco, CA, USA).

### 3.7. The Determination of NO Production from RAW 264.7

To evaluate the effective of the tested materials on LPS-induced NO production, Raw 264.7 macrophages in 10% FBS-DMEM were plated at densities of 1.0 × 10^5^ cell/wells in 96-well plates and grew overnight, after incubation, 20 μL serially diluted drugs (DMSO+serum-free DMEM as solvent) were applied to the cells for 4 h, and incubated in the medium with 20 μL LPS (lipopolysaccharide) of 1 μg/mL in the presence or absence of test samples for 24 h. Then, cells were dispensed into 96-well plates. One hundred μL of each supernatant was mixed with the same volume of Griess reagent [28] (1% sulfanilamide in 5% H_3_PO_4_ and 0.1% *N*-1-naphthyletylenediamide dihydrochloride) and incubated at room temperature, away from the light, for 10 min. The absorbance was measured at 540 nm using an ELISA reader (PerkinElmer, Waltham, MA, USA), and the concentration of nitrite was calculated by comparison with a sodium nitrite standard curve. For this assay, *N*-monomethyl-l-arginine (l-NMMA) was used as positive control. Three independent experiments were performed, with each one in triplicate.

### 3.8. Statistical Analysis

The IC_50_ values, the sample concentrations resulting in 50% inhibition of NO production, were determined by using nonlinear regression analysis (Sigma Plot 8.0; SPSS Inc. Chicago, IL, USA). The data are presented as mean ± S.D. of more than three independent experiments. 

## 4. Conclusions

This study demonstrated that the leaves of *D. metel* were rich in withanolides, showing a similar structure as compounds found in its flowers. Moreover, five withanolides, including four new compounds **1**–**4**, were isolated and identified, and these compounds were found to be responsible for the ability to inhibit NO production by activated macrophages. According to our results, further phytochemical and pharmacological studies of leaves *D. metel* are clearly worthwhile.
